# Determinants and predictive modelling of barriers to HIV testing uptake among adolescents and young adults in a private higher education institution in Botswana

**DOI:** 10.1371/journal.pone.0352165

**Published:** 2026-06-26

**Authors:** Gabalape Arnold Sejie, Thato Lebang, Gosego Ngole, Onneile Dichabe, Merapelo Selwane, Oratile Marapo, Kefilwe Madikwe, Goratang Malgas, Tumelo Ikaneng

**Affiliations:** 1 School of Public Health, Boitekanelo College, Gaborone, Botswana; 2 Department of Health Promotion and Education, Boitekanelo College, Gaborone, Botswana; Institute of Tropical Medicine / University of Antwerp, BELGIUM

## Abstract

**Background:**

HIV represents a significant public health challenge, contributing to increased mortality and morbidity in developing countries, particularly Sub-Saharan Africa. While HIV testing is crucial for early treatment and prevention, the uptake of the HIV testing among the adolescent and young adults in Botswana remains low. This study aims to predict barriers to HIV testing uptake among adolescents and young adults.

**Method:**

A quantitative cross-sectional study was conducted. Data was obtained using a questionnaire employing a simple random sampling technique to collect the survey data. Statistical analysis involved descriptive statistics and multivariable logistic regression.

**Results:**

A total of 353 participants were recruited. The prevalence of HIV testing uptake in the preceding 12 months was (64.9%; n = 229). Determinants associated with non-uptake of HIV testing included participants in casual relationships (OR 1.776,95%CI 0.998–3.160, P < 0.049), transactional relationships (AOR 1.098, 95% CI 0.189–6.382) compared with single participants. Additional determinants included residence beyond 5 km from HIV testing centers (OR 1.825, 95% CI 1.074–3.103, P < 0.026), and cohabiting (AOR 3.45, 95% CI 0.271–43.979). Religious affiliation was also predictive, with Christians (AOR 2.347, 95% CI 0.188–29.295), Muslims (AOR 1.765, 95% CI 0.046–59.506), and adherents of African traditional religions (AOR 1.718, 95% CI 0.084–35.004) exhibiting higher odds of non-testing compared with non-believers. Participants with negative attitudes toward HIV testing were 69% less likely to forgo HIV testing (OR 0.310, 95% CI 0.1704–3.103, P = 0.026) than those with positive attitudes.

**Conclusion:**

This study provides critical evidence on disparities in HIV testing among adolescents and young adults in Botswana, highlighting persistent gaps in access and utilization. These findings underscore the necessity for context-specific strategies that mitigate access barriers and address behavioral, structural, and socio-cultural determinants to optimize HIV testing uptake, advancing progress toward the UNAIDS 2030 target of zero new infections.

## Introduction

Human Immunodeficiency Virus (HIV) remains a significant global public health concern with an estimated 40.8 million individuals reported living with HIV in 2024 [1]. The World Health Organization (WHO) African Region inhabited by 12% of the world’s population accounted for over two thirds of the global HIV burden [1]. In addition, approximately 630, 000 lives were lost due to HIV-related causes worldwide with 97% of these deaths occurring in developing nations, Sub-Saharan Africa being the primary region carrying a disproportionate burden [1]. As a result, the Joint United Nations Programme on HIV/AIDS (UNAIDS) has considered an acceleration of response aimed at eliminating HIV by the year 2030. To achieve this, the 95-95-95 targets was formulated [2]. This target aims to ensure that, by 2030, 95% of people living with HIV know their serostatus, 95% of people diagnosed as HIV-positive receive antiretroviral treatment, and 95% of people on antiretroviral treatment have their viral load sustainably suppressed. Thus, access to screening remains the main element to be taken into account to achieve this objective [2]. HIV testing is therefore a fundamental approach that serves as a crucial initial step in the continuum of HIV care enabling individuals to become aware of their HIV status and to seek treatment and/or preventive care [2,3]. In 2024, the global HIV response was closer than ever to reaching these testing and treatment targets. Globally, an estimated 87% (69– > 98%) of all people living with HIV knew their HIV status, 89% (71– > 98%) of people who knew their HIV-positive status were receiving antiretroviral therapy, and 94% (75– > 98%) of people on treatment had a suppressed viral load [1].

While global trends show an increase in HIV prevalence and significant declines in AIDS-related deaths, largely due to the increase of antiretroviral treatment (ART), Botswana a country endemic to HIV with a prevalence of 20.8% exceeded all UNAIDS 95-95-95 targets at 95.1%, 98.0%, and 97.9% among adults (15–64 years) living with HIV. However, while the country’s overall goals are being met, the rates of HIV testing among those aged 15–24-year-old are concerning, as many young people particularly young women are unaware of their HIV stats with only 85% diagnosed (15% Unaware of their status), 83% on treatment, and 76% virally suppressed indicating a lag behind among this age group [4]. Additionally, a total of 4,120 people contracted new HIV infections in Botswana in 2024, with 27 percent of them reported to be adolescent girls aged between 15 and 24 [5]. This lack of epidemic control could be attributed to the low HIV testing participation among these age groups. This is substantiated by the fact that a relatively low proportion of young people received an HIV test within a 12-month period and that young people made up the largest proportion of those who tested positive that were unaware of their status [4]. This demographic is primarily composed of tertiary institutions, with Boitekanelo college being one of the private tertiary institutions in Botswana, hosting over 3000 students. However, no study has been conducted to identify predictors of HIV testing uptake, highlighting a gap in knowledge and population understanding in this area of research thus the aim/purpose of the study was to identify and predict barriers to HIV testing among adolescents and young adults in a private tertiary institution in Botswana.

## Methods

### Study design and setting

A quantitative cross-sectional descriptive study with an analytical component was conducted in Boitekanelo college, Tlokweng, Masetlheng ward, Botswana with geographical coordinates 24.6872° S, 25.9783° E. The college is situated approximately 10.6 km east of Gaborone accessible by A12 road enroute to the Tlokweng boarder post to South Africa. Boitekanelo College is a healthcare education institution offering a variety of health programs with just above 3000 undergraduate student population.

### Selection of study population

The study population included adolescents and young adults aged less than 24 years studying full-time at Boitekanelo college. Adolescent students without Parental Consent, unwilling to participate and those who were pregnant were excluded.

### Sampling

The study employed a simple random sampling strategy calculated using the Yamane formular at a 95% confidence level and a 5% margin of error to get an ideal participant sample size of 356 participants for the study from a total population of 3266.

### Data collection procedure

Participants were located using their contacts from the admissions registers, following exclusions, a self-administered structured questionnaire was then utilized to gather data from 22 November 2025–27 November 2025.

### Study variables

#### Outcome variables.

The outcome variable was HIV testing Uptake, which is a single direct question asking whether the respondent has tested within the preceding 12 months. The response for this variable was binary which was coded as “0” for no and “1” for yes based on their self-reported HIV testing status.

#### Independent variables.

Individual independent variables inclusive of demographic information such as Gender, Age, Religion, Education, Occupation Status, Marital Status, Distance, Stigmatizing Attitudes, Risky Behavior, Level of HIV/AIDS testing knowledge were studied. The knowledge variable was operationalized as a composite index capturing participants’ understanding of HIV transmission pathways, prevention modalities, and awareness of HIV testing services, including where such services can be accessed with a common scoring method used for knowledge questions [6,7] as follows: 1 point was given for correct answers, and 0 for incorrect. A participant who scored less than 50% correct answers was considered as having poor knowledge, 50–80% considered as fair knowledge, and those who scored at least 80% were considered to be having good knowledge. The attitude variable was measured as a multidimensional construct capturing respondents’ positive or negative dispositions toward HIV testing. It was assessed through items examining beliefs about the perceived benefits of knowing one’s HIV status such as improved access to treatment and psychological reassurance as well as affective responses to HIV testing, related services, and living with HIV. Responses were aggregated to generate an overall attitude score, with higher values indicating more favorable attitudes toward HIV testing. The degree of the estimation through the values of the resulted means, were encoded to the Likert scale as follows; scores between 1.0–2.4 indicate a negative attitude, scores between 2.5–3.4 reflect a neutral attitude, and scores between 3.5–5.0 indicate a positive attitude [8,9].

### Pretesting

The questionnaires were developed and translated into the native language being Setswana. The tools were pretested on 35 participants (10% of our total population) by well-trained research assistants. The principal investigator also checked data for completeness. This provided valuable insights into the feasibility, effectiveness, and potential challenges of implementing large-scale research and the tool was adjusted to improve its reliability and validity.

### Measures to reduce selection and information bias

The study participants were selected using specific inclusion criteria for the study. Sampling bias was reduced by matching participants sampling frame to the target population.

### Data analysis

Data was cleaned to reduce inconsistencies and analyzed using SPSS software version 30. The analysis was carried out using a set of statistical methods (i.e., descriptive statistics and multivariable logistic regressions). Descriptive analysis was employed to describe characteristics of individual related factors using frequencies, measures of central tendency and variation, proportions, and numerical summary measures. An adjusted odds ratio (AOR) with a corresponding 95% confidence interval (CI) was computed and reported. Statistical significance was declared at p-value *<* 0.05. Variables were selected a priori based on theoretical relevance and prior literature, and all were included in the model regardless of p-values to ensure adequate control of potential confounding and to obtain adjusted estimates reflecting the independent contribution of each variable.

### Ethical approval

The study obtained ethical approval from the Botswana Health Research and Development Division dated November 20, 2025 (reference number: HPRD 6/14/1) before the study was conducted. Permission was obtained from the study site. The study has been planned according to the declaration of Helsinki (last updated; October 2013) which is as a statement of ethical principles for medical research involving human participants, including research using identifiable human material or data.

### Informed consent

The consent form was read and signed by study participants. The consent form included contact information for the researcher and the research ethics committees that approved the study as well as a full description of the study objective. The anonymity and confidentiality of the obtained data were always assured.

## Results

### Study population

Three hundred and fifty-six participants were eligible for inclusion in the study. Four did not consent; therefore, only 352 participants were included in the analysis. The survey response rate measured by dividing the total number of survey responses by the number of invitations sent to potential participants (sample size) was 98.9%.

### Socio-demographic profile of the participants

The study population had a mean age of 20.78 years (Standard deviation (SD):1.539) and a median age of 21 years (IQR: 20–22 years). On measures of symmetry, age mean, median, and mode showed a zero-skewness suggesting a perfectly symmetrical distribution. Over three quarter of the respondents, were in the age category of 20–24 accounting for 75.9% (n = 268) with females making up more than two third of the respondents (n = 243; 68.8%). On marital status, single participants made up the largest population (77.1%; n = 272) and more than two thirds (n = 223) of the participants travelled less than 5 km to access HIV testing centers. Public transport was the most popular form of transportation (44.5%; n = 157) followed by those walking with 51.8% (n = 183) least being those who travelled using private vehicles at 3.7%(n = 13). Christians dominated the religious spectrum with 95.1%(n = 336), those with moderate knowledge on HIV testing services constituted 87.8%(n = 310) while over one third of the participants reported to be having negative attitude (36.5%; 129), neutral attitude (39.1%;138) and positive attitude (24.2% (86%) towards HIV testing. On access to HIV testing information, 69.7% reported to have heard of it from school, health facilities (n = 54;15.3%), social media(n = 33;9.3%) and the least with 5.7%(n = 20) reported hearing about it from friends and families. Almost three fifths of the participants reported-risk behavior of unprotected sex (41.1%; n = 145), multiple concurrent partnership (5.9%; n = 21), touching blood (4.5%; n = 16) and a relatively small percentage sharing needles (1.1%; n = 4). (**Table 1**).

**Table 1:** Socio demographic characteristics of survey respondents.

Variable	Subcategories	Frequency (n)	Percentage (%)
Age	15-19	85	24.1
	20-24	268	75.9
Gender	Male	106	30
	Female	243	68.8
	Prefer Not To Say	4	1.1
Religious Belief	Christian	336	95.2
	Muslim	3	0.8
	African Traditional Religion	11	3.1
	Atheist	3	0.8
Marital Status	Single	272	77.1
	Cohabiting	77	21.8
	Married	4	1.1
Type Of Relationship	Peer	158	44.8
	Transactional	10	2.8
	Intergenerational	9	2.5
	Casual	21	5.9
	Single	155	43.9
Mode Of Transport	Walking	157	44.5
	Public Transport	183	51.8
	Private Cars	13	3.7
Distance	>5km	130	36.8
	<5km	223	63.2
Cues To Action for HIV Testing (Internal or external triggers that prompt an individual to get tested)	School	246	69.7
	Clinic/Hospital	54	15.3
	Social media	33	9.3
	Friends Or Family	20	5.7
HIV Knowledge Level	Poor	3	0.8
	Moderate	310	87.8
	Excellent	40	11.3
HIV Testing Attitudes (Positive Or Negative Dispositions Toward HIV Testing)	Negative	129	36.5
	Neutral	138	39.1
	Positive	86	24.4
HIV status	Positive	6	1.7
	Negative	223	63.2
	Unknown	124	35.1
Perceived Risky Behaviours	Multiple Concurrent Partners	21	5.9
	Unprotected Sex	145	41.1
	Sharing Needles	4	1.1
	Touching Blood	16	4.5
	Not Exposed To Risky Behaviors	167	47.3

### Outcome descriptive

Regarding HIV testing uptake, approximately two-thirds of participants (64.9%; n = 229) reported prior HIV testing within the preceding 12 months. Among those tested, 97.4% (n = 223) were seronegative. **See Table 1**.

### Multivariable predictive modelling of barriers to HIV testing

Our study showed that participants who were engaged in casual relationships were 1.7 times significantly likely to not undergo HIV testing compared to those not in any relationship (AOR 1.776,95%CI 0.998–3.160, P < 0.049). The likelihood of not undergoing HIV testing was significantly higher among participants residing more than 5 km from HIV testing centers (AOR 1.825, 95% CI 1.074–3.103, P = 0.026) compared to those living within 5 km. Participants with negative attitudes toward HIV testing were 69% less likely to forgo HIV testing than those with positive attitudes (AOR 0.310, 95% CI 0.1704–3.103, P = 0.026). Age was also associated with testing behavior, with individuals aged 15–19 years exhibiting higher odds of not testing compared to those aged 20–24 years (AOR 1.607, 95% CI 0.894–2.888). Religious affiliation influenced testing uptake: participants identifying as Christian (AOR 2.347, 95% CI 0.188–29.295), Muslim (AOR 1.765, 95% CI 0.046–59.506), adherents of African traditional religions (AOR 1.718, 95% CI 0.084–35.004) were more likely not to take an HIV test compared to non-believers. Marital status and relationship type were also relevant, with cohabiting (AOR 3.45, 95% CI 0.271–43.979), single (AOR 2.018, 95% CI 0.166–24.499), and participants engaged in transactional relationships (AOR 1.098, 95% CI 0.189–6.382) demonstrating higher odds of not taking an HIV test in the preceding year. Additionally, sources of HIV testing information impacted uptake; participants who received HIV testing information via social media (AOR 3.334, 95% CI 0.755–14.721), school (AOR 1.878, 95% CI 0.508–6.945), or health facilities (AOR 1.714, 95% CI 0.273–4.767) were more likely not to undergo HIV testing. **See Table 2**.

**Table 2:** Multivariable logistic regression analysis of factors associated with non-uptake of HIV testing among adolescents and young adults.

Variable	Subcategories	AOR	95% Confidence Interval		P Value
			Lower Bound	Upper Bound	
Age	15-19	1.607	0.894	2.888	0.113
	20-24	.	.	.	.
Gender	Male	0.489	0.035	6.822	0.595
	Female	0.326	0.024	4.447	0.401
	Prefer not to say	.	.	.	.
Religious belief	Christian	2.347	0.188	29.295	0.508
	Muslim	1.647	0.046	59.506	0.785
	African Traditional Religion	1.718	0.084	35.004	0.725
	Atheist	.	.	.	.
Marital status	Single	2.018	0.166	24.499	0.581
	Cohabiting	3.45	0.271	43.979	0.34
	Married	.	.	.	.
Type of relationship	Peer Relationship	0.467	0.087	2.503	0.374
	Transactional relationship	1.098	0.189	6.382	0.917
	Intergenerational relationship	0.53	0.161	1.742	0.296
	Casual relationship	1.776	0.998	3.16	0.049
	None	.	.	.	.
Modes of transport to access to HIV testing services	Walking	0.852	0.498	1.455	0.557
	Public transport	0.682	0.166	2.807	0.596
	Private car				
Distance to testing facility	>5KM	1.825	1.074	3.103	0.026
	<5KM	.	.	.	.
Cues to action for HIV testing (Internal or external triggers that prompt an individual to get tested)	School	1.878	0.508	6.945	0.345
	Clinic/Hospital	1.14	0.273	4.767	0.857
	Social media	3.334	0.755	14.721	0.112
	Friends or family	.	.	.	.
HIV knowledge level	Poor	0.593	0.025	14.024	0.746
	Moderate	0.916	0.429	1.956	0.822
	Excellent	.	.	.	.
HIV testing attitudes (positive or negative dispositions toward HIV testing)	Negative	0.31	0.161	0.597	<.001
	Neutral	1.045	0.571	1.912	0.887
	Positive	.	.	.	.
Risky behaviors	Multiple concurrent partners	0.828	0.286	2.399	0.728
	Unprotected sex	0.722	0.405	1.289	0.271
	Sharing needles	0.639	0.141	2.891	0.561
	Touching blood	1.146	0.391	3.365	0.804
	Not exposed to risky behaviors				.

## Discussion

HIV testing is a fundamental strategy that acts as an essential first step in the continuum of HIV care, allowing individuals to be aware of their HIV status and seek treatment and/or preventive care [2,3]. However, Botswana’s HIV testing rates among those aged 15–24-year-old are alarming, with a substantial proportion of young people remaining untested, hampering the national and global prospects of ending HIV by 2030. We assessed predictors of barriers to HIV testing uptake among this age group which is critical for the effectiveness of HIV programs.

Approximately one-third (35%) of the youths in this study had not undergone HIV testing in the preceding year and were therefore unaware of their HIV status. This finding is consistent with the finding from other studies done in Ethiopia [10] and a demographic and Health Survey (DHS) data from four geographic regions of sub-Saharan Africa where 34.9% and 36.5% of youths were ever tested for HIV [11]. The study also revealed a notable difference in the HIV testing uptake among young people who live varying distances from HIV testing facilities. Young people who travelled more than 5 km were positively associated with the likelihood of not receiving an HIV test in the past 12 months. These findings expand on prior research that suggests an association between traveling a longer distance to HIV testing sites and lower likelihood of getting tested for HIV [12]. This is further corroborated by a nested, explanatory-sequential study in Uganda which revealed that, young people living >10km from the nearest HIV testing facility were less likely to have tested than those who lived closer to it (<5km) [13]. Previous studies have also found that distance restricts young people from accessing health care services including HIV testing services [14] suggesting that the distance required to travel to receive HIV screenings possibly due to living in areas with lower provider density, resulting in reduced frequency of HIV testing thus a major barrier to testing in this population [12].

Interestingly, this study also found that participants holding negative attitudes towards HIV testing were less likely to forgo HIV testing. This is contrary to the current literature, which states that more negative attitudes are associated with refusal of an HIV test or never having had an HIV test in sub-Saharan Africa [15], and that accepting an HIV test is associated with more positive attitudes towards testing [15]. This may be due to the fact that overall negative attitude score does not account for the weight of these individual attitudes in their decision making. However, this does not mean that these factors are not important considerations when planning to implement a testing program, they remain a fundamental part of the HIV testing process and likely play a role in the complex decision-making process around testing acceptance.

Adolescents had higher odds of not undergoing HIV testing than young adults. This concurs with previous studies showing increasing age associated with testing [16]. This is corroborated by an explanatory sequential study in Uganda that young people aged 20–24 years were more likely to test for HIV than those aged 15–19 year [13]. Possible explanations could be that young adults are more knowledgeable about the risks and importance of testing, especially since they are more likely to be sexually active or have more sexual experience than adolescents and age of consent to testing laws and that they have more autonomy and decision-making powers [16]. However, these findings are contrary to a cross-sectional study conducted in Uganda which found that adolescents aged 15–19 years had higher odds of HIV testing [17].

Our study also found an increased odds for not taking an HIV test among risky sexual behavior categories like casual and transactional relationships, this is similar to a study in western Kenya showing that HIV testing use was lower among partners who engaged in casual or transactional sex with an index client, compared to sexual encounters within relationships [18]. Additionally, a cross-sectional population-based survey conducted in KwaZulu-Natal, South Africa found people with higher risk sexual behavior to be significantly less likely to know their HIV-positive status and, among those aware, less likely to be virally suppressed [19]. This could be explained that risky behavior clients are likely to test for HIV primarily due to perceived low personal risk, fear of stigma and potential results, and a lack of consistent, accessible testing, all of which contribute to them not seeking testing services. They may also underestimate the prevalence of HIV among their peer groups.

Majority of participants in the current study discovered HIV testing at school, meaning that HIV testing information is given more at a school setting than other settings. This is supported by a systematic review and meta-analysis study conducted in sub-Saharan Africa pronouncing that, sexual health education delivered in a school setting aims to promote sexual health by ensuring that they educate students on the HIV testing uptake [20] therefore, those that are not attending school stand at a high chance of not being aware of HIV testing thus the low uptake of HIV testing among them [20]. Adolescents and young adults’ religions were also independently predicted their HIV test uptake; for Christianity, Muslim and African traditional religion followers, the odds of undergoing an HIV test were lower compared to non-believer youths. This is in concordance with an Ethiopian youth study revealing that, Muslim and other religion follower youths’ odds of getting HIV test were lower [10]. Additionally, this finding supports the finding from the study done among reproductive age Ethiopian women where Christian and Muslim women were less likely to accept HIV testing [21]. A possible explanation could be that shame-related HIV stigma is strongly associated with religious beliefs such as the belief that HIV is a punishment from God or that people living with HIV/AIDS (PLWHA) have not followed the Word of God [10]. Thus, the differences in beliefs and dogmas across different religions may be playing a role.

The likelihood of taking an HIV test was lower among single and cohabiting relationships compared to the married. This concurs with a cross-sectional retrospective analysis of HIV programs in Rwanda showing that, the likelihood of being tested was lower among cohabiting relationships compared to other contact-index relationships such as unknown/occasional sex partners, children, boyfriends or girlfriends, and others [22]. This could be due to the nature of the relationship between the index case and the contact, which is likely to be a single or cohabiting partner in cases with one or two contacts in the last 3 months. This relationship may lead the index partner to fear disclosing their HIV status to their partners or fear of stigma among single index clients and thus, reduce their chance of being tested for HIV [22]. Additionally, another study revealed that married youths and youths with another form of marital status, were nearly 5.37 and 4.60 times likely to get HIV test compared to their peer youths who are not in marital union [10]. Studies from Malawi [23], and Senegal [24] using secondary DHS data analyses also showed that ever-married women more likely accept HIV testing as compared to never-married women which are consistent with the findings from the current study.

### Limitations of the study

Although this study builds on the existing literature on HIV testing, several limitations were encountered which should be considered when interpreting the findings. One limitation was the cross-sectional nature of the data limiting the determination of the temporal nature of the associations reported in this study. The outcome variable was measured by self-reporting; as such, social desirability and recall bias cannot be excluded entirely, though this is a common phenomenon it may affect the accuracy of the data.

## Conclusion

This study highlights the need for health policy makers and other stakeholders to sustain current efforts by targeting adolescent’s and younger people aged 15–24 with customised interventions to address disparities in HIV testing uptake. These tailored interventions addressing key factors are crucial for enhancing testing accessibility, emphasizing awareness campaigns, proximal HIV testing service access, and targeted behavioural and education efforts. Targeting these groups in a timely manner is a crucial and necessary step not only for achieving the UNAIDS’ 95–95−95 targets but for individuals to benefit from early diagnosis, treatment and HIV prevention efforts to sustainably reduce population level HIV transmission.

### Recommendation

A further qualitative study would be necessary to assess the attitudes, values and beliefs held by participants which could potentially affect their HIV testing uptake. This will provide in-depth explanations of the quantitative results enabling researchers to delve deeper into the “why” and “how” behind the current quantitative study results, potentially identifying new variables or clarifying existing relationships behind HIV testing uptake thus providing richer context and helping to understand the nuances of the quantitative results.

## Abbreviations

AOR: Adjusted odds ratio
ART: Antiretroviral treatment
CI: Confidence interval
DHS: Demographic and Health Survey
HIV: Human Immune deficiency Virus
IQR: Inter Quartile Range
PLWHA: People living with HIV/AIDS
UNAIDS: United Nations Programme on HIV/AIDS
WHO: World Health Organization
